# RETRACTED: Aldawsari et al. Gum Acacia Functionalized Colloidal Gold Nanoparticles of Letrozole as Biocompatible Drug Delivery Carrier for Treatment of Breast Cancer. *Pharmaceutics* 2021, *13*, 1554

**DOI:** 10.3390/pharmaceutics16060721

**Published:** 2024-05-27

**Authors:** Hibah M. Aldawsari, Sima Singh, Nabil A. Alhakamy, Rana B. Bakhaidar, Abdulrahman A. Halwani, Shaimaa M. Badr-Eldin

**Affiliations:** 1 Department of Pharmaceutics, Faculty of Pharmacy, King Abdulaziz University, Jeddah 21589, Saudi Arabia; nalhakamy@kau.edu.sa (N.A.A.); rbakhaidar@kau.edu.sa (R.B.B.); aahalwani@kau.edu.sa (A.A.H.); 2 Center of Excellence for Drug Research and Pharmaceutical Industries, King Abdulaziz University, Jeddah 21589, Saudi Arabia; 3 IES Institute of Pharmacy, IES University Campus, Bhopal 462044, India; simasingh87@gmail.com; 4 Mohamed Saeed Tamer Chair for Pharmaceutical Industries, Faculty of Pharmacy, King Abdulaziz University, Jeddah 21589, Saudi Arabia

The *Pharmaceutics* Editorial Office retracts the article, “Gum Acacia Functionalized Colloidal Gold Nanoparticles of Letrozole as Biocompatible Drug Delivery Carrier for Treatment of Breast Cancer” [1], cited above.

Following publication, concerns were brought to the attention of the publisher regarding the overlap of images.

Adhering to our complaint procedure, an investigation was conducted that confirmed a partial duplication between Figures 5b and 5e, presenting different experimental conditions.

While the authors fully cooperated with the Editorial Office during the investigation, they were unable to satisfactorily explain the overlap of figures and meet the required quality standards of raw images in order to consider a correction as per the journals original image requirements policy (https://www.mdpi.com/journal/pharmaceutics/instructions#oriimages). As a result, the Editorial Board and Editor-in-Chief were unable to confirm the reliability of the findings and subsequently decided to retract this paper as per MDPI’s retraction policy (https://www.mdpi.com/ethics#_bookmark30) and in line with the Committee on Publication Ethics retraction guidelines (https://publicationethics.org/retraction-guidelines). 

This retraction was approved by the Editor-in-Chief of the journal *Pharmaceutics*.

The authors did not agree to this retraction. 
